# Contrasting life history in the diminutive *Dimetrodon* species from North America and Germany

**DOI:** 10.1038/s41598-026-52199-y

**Published:** 2026-06-16

**Authors:** Aurore Canoville, Philipp L. Knaus, Lorenzo Marchetti, Jörg Fröbisch

**Affiliations:** 1 Museum für Naturkunde Leibniz-Institut für Evolutions- und Biodiversitätsforschung, Berlin, Germany; 2 North Carolina Museum of Natural Sciences, Raleigh, NC USA; 3 Friedenstein Stiftung Gotha, Gotha, Germany; 4 Humboldt-Universität zu Berlin, Berlin, Germany

**Keywords:** Bromacker locality, Sphenacodontia, Paleohistology, Growth strategy, Paleoenvironment, Dwarfism, Ecology, Ecology, Evolution, Zoology

## Abstract

The sphenacodontid *Dimetrodon* is best known from lower Permian marginal-marine deposits of North America (NA), where it occupied apex predator roles in aquatic-based food chains. *Dimetrodon teutonis*, the smallest documented species and only known representative of this genus outside NA, comes from the fully terrestrial and resource-limited ecosystem of the upper Asselian Bromacker locality, Germany. To evaluate its growth strategy and diminutive nature, we present the first osteohistological analysis of *D. teutonis*. We also reassess NA material from four localities spanning distinct depositional settings. In contrast to the thick and highly vascularized woven to parallel-fibered bone cortices of most NA species, including the small-bodied *D. natalis*, *D. teutonis* exhibits thin cortices composed of poorly vascularized parallel-fibered bone. The presence of an external fundamental system indicates somatic maturity and confirms its diminutive nature. These data suggest extended and slow growth throughout ontogeny, likely linked to seasonal resource limitation and low predation pressure in the unique Bromacker ecosystem. In contrast, NA diminutive species inhabiting lowland ecosystems grew rapidly and matured early, in response to competition and high predation pressure. These contrasting life histories in small *Dimetrodon* species reflect differing ecological pressures and illustrate diverging evolutionary pathways to dwarfism in this iconic genus.

**Supplementary Information:**

The online version contains supplementary material available at 10.1038/s41598-026-52199-y.

## Introduction

About a dozen species ascribed to the iconic sail-backed sphenacodontid *Dimetrodon* have been reported from Cisuralian (lower Permian) North American (NA) fossil deposits (i.e., in the southwestern United States and Atlantic Canada;^1–4^). Their remains are most abundant in localities of Texas and Oklahoma that share similar fossil assemblages. These paleoecosystems represented marginal-marine deltaic, floodplain, and swampy environments with perennial rivers, abundant vegetation, and mixed vertebrate faunas of aquatic to terrestrial taxa^5,6^. In these tetrapod communities, herbivores were relatively scarce, while medium to large-sized carnivorous amniotes, including some *Dimetrodon* species and other early synapsids, dominated the trophic pyramid in terms of relative abundance^7^. In North America, *Dimetrodon* species ranged in adult total body length and body weight from about 1.7 m and 38 kg for the diminutive and basal *D. natalis*, up to 4.6 m and 250 kg for *D. angelensis*^1,2^. Fossil occurrences in some of these localities suggest sympatric *Dimetrodon* species of different body sizes that may have occupied distinct ecological niches^8–10^. Although *Dimetrodon* is well adapted to a terrestrial lifestyle, it has often been hypothesized as fully dependent on an aquatic vertebrate-based diet^11,12^ and thus constrained to habitats near persistent water bodies. However, a few isolated reports of this genus in more inland environments, distant from permanent aquatic systems, such as the Abo Formation in New Mexico^13^ or the Richards Spur locality in Oklahoma^4,14^, suggest that these animals may have adapted to a wider range of ecological settings. Notably, *Dimetrodon* species found in the latter localities (i.e. *Dimetrodon occidentalis* and a yet unnamed species) represent rather small-bodied forms of the genus^4,13^.

*Dimetrodon teutonis* represents the only confirmed species of this genus outside North America and is reported from the lower Permian (upper Asselian) Bromacker locality of the Tambach Formation, central Germany^2,12,15^. Interestingly, it is considered the smallest *Dimetrodon* species ever discovered, with an estimated snout-vent length of 55 cm^12^ and a body mass of 24 kg^2^ based on its vertebral centrum width (method following^1^. Its diminutive size has been hypothesized as an adaptation to a specific paleoenvironment^12^. Indeed, the paleoecosystem preserved at Bromacker was inland, possibly isolated with a rather endemic fauna, and characterized by ephemeral water bodies^7,16,17^. It thus differed substantially from most coeval European lacustrine and NA marginal marine assemblages, with the Richards Spur locality being a notable exception^4,14^. The Bromacker paleoclimate was highly seasonal with extended dry phases^17,18^. As a result, its tetrapod fauna was exclusively terrestrial, with abundant herbivores, but scarce and small to medium-sized predators^7^. Finally, at Bromacker and Richards Spur, unlike in lowland NA localities, some medium-sized varanopids shared the top predator niche with small *Dimetrodon* species^2,4,14,19^. These predators likely subsisted on small vertebrates that were abundant in these localities^12,20^.

Environmental conditions have been documented to impact the growth strategies and life history of vertebrates (i.e^21,22^), however this has been understudied in late Paleozoic tetrapod communities. Few studies have examined the osteohistology of NA *Dimetrodon* species, mostly focusing on axial elements, and in particular the hyperelongated neural spines holding the dorsal sail of these animals (e.g.^4,23–28^). These bones show a figure-8 or dumbbell cross-section characteristic of this genus^4,25,26^. In medium to large-sized species, lateral cortices are thick and composed of highly vascularized, sometimes woven-fibered, zones with longitudinal primary osteons often organized in radial rows^25,26^. Interestingly, Brink et al.^4^ sampled the neural spine of a potentially small *Dimetrodon* species from the Richards Spur locality and noted it had less vascularized cortices, no woven tissue, and fewer remodeling than larger forms. They hypothesized that such neural spine microstructure might be typical of small-bodied species^4^.

Ricqlès^25^ was the first to investigate *Dimetrodon* long bone histology based on different-sized, but unidentified species from NA localities. Huttenlocker & Rega^27^ sampled a single tibia of *D. giganhomogenes* from the Arroyo Formation. Finally, Shelton et al.^9^ examined the limb bone histology of species from the Briar Creek Bonebed (Nocona Formation), including a humeral and femoral growth series of the diminutive *D. natalis.* These studies congruently reported on the presence of compact stratified cortices with an alternation of avascular and relatively thin annuli (some coupled to lines of arrested growth - LAGs) made of a parallel-fibered to lamellar matrix, and well- to highly-vascularized thicker zones usually composed of parallel-fibered tissue. The vascularization pattern is rather homogenous in NA members of the genus and consists of longitudinal primary osteons arranged in radial rows and often interconnected by radial anastomoses. Simple oblique or radial vascular canals are also frequent^9,25,26^. *D. natalis* possesses relatively thick cortices and the presence of woven tissue coupled to numerous primary osteons in its zones is reminiscent of the woven-parallel complex and suggests a general high growth rate in this diminutive species^9^. Cortical remodeling is always very limited and restricted to the endosteal margin occupied by large sub-circular erosion bays. Secondary osteons are never found in the rest of the cortex. Finally, the medullary cavity is usually filled by a spongiosa in these animals, even at the mid-diaphyseal level.

In the present study, we investigate for the first time the long bone and neural spine microstructure of *D. teutonis*, the only *Dimetrodon* species recovered outside NA, and additional comparative NA material with the aims to:

i) test the hypothesis that *D. teutonis* represents a dwarf species, including a reassessment of its body mass based on stylopodial circumferences^29^;
ii) compare the bone microanatomy and growth patterns of *D. teutonis* with those of NA species, notably the other confirmed diminutive species, *D. natalis*;
iii) explore the influence of environmental settings (including climate, resource availability, predation and competition pressures) on growth strategies and physiological adaptations in these early Permian tetrapods.

Given the contrasting depositional and paleoecological contexts of the Bromacker and most NA localities, this comparative histological analysis offers a novel opportunity to examine how environmental pressures shaped the life history and morphology of one of the most iconic Permian predators.

## Institutional abbreviations

**IPBSH**: Paleohistology collection of the former Steinmann Institute of Geology, Mineralogy and Palaeontology, University of Bonn, Bonn, Germany (all IPBSH sections are now curated at the Museum für Naturkunde, Berlin, Germany); **MB.R.**: Museum für Naturkunde, Berlin, Germany; **MNG**: Friedenstein Stiftung Gotha, Gotha, Germany; **OMNH**: Sam Noble Museum, University of Oklahoma, Norman, Oklahoma, USA; **TMM**: Texas Memorial Museum, Jackson School of Geosciences; University of Texas, Austin, Texas, USA.

## Results

**Fig. 1:**
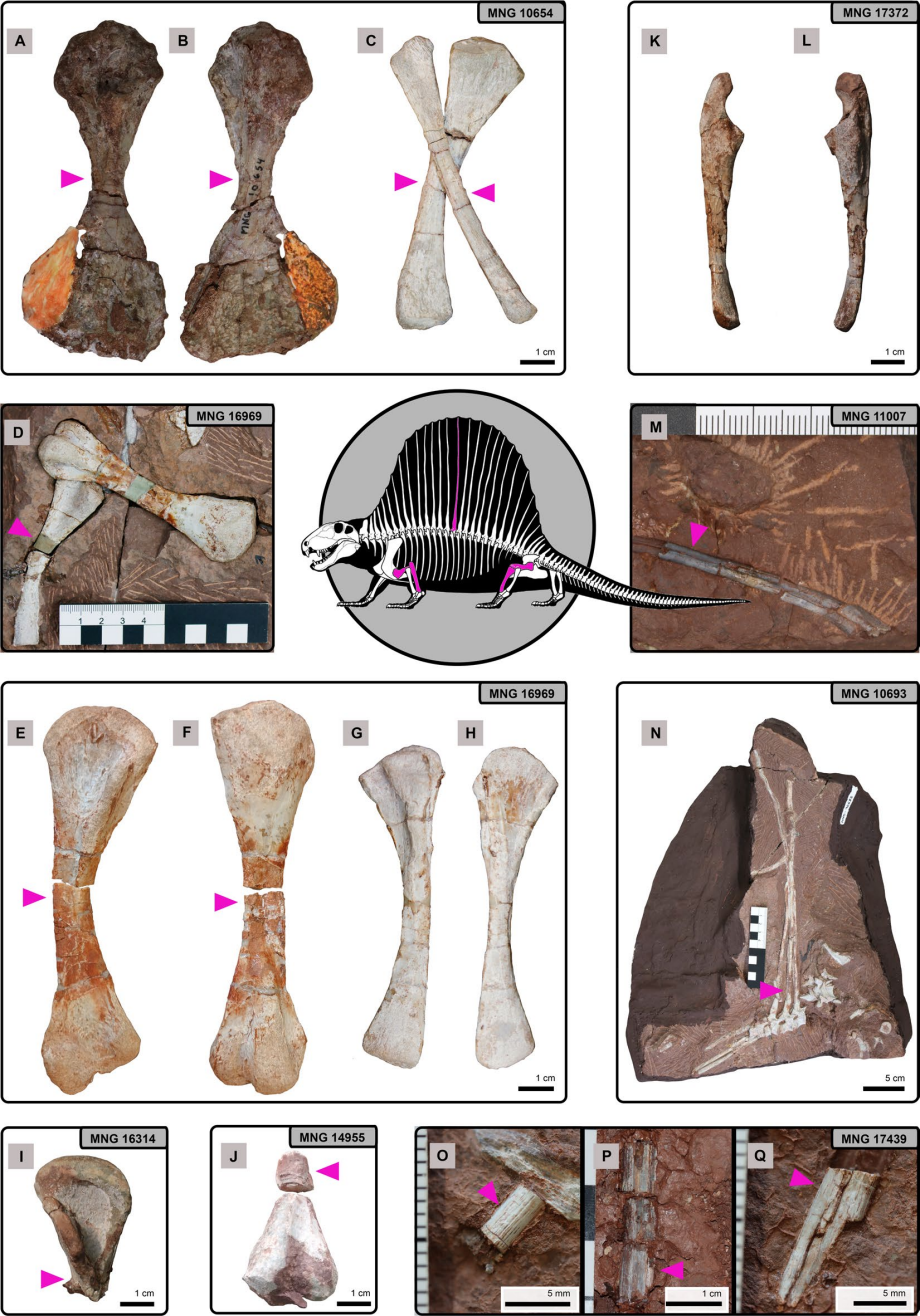
*Dimetrodon teutonis* material investigated in this study. (**A**) MNG 10654 left humerus in ventral view; (**B**) same as (**A**) in dorsal view; (**C**) MNG 10654 left tibia and fibula in dorsal view; (**D**) MNG 16969 left femur and tibia in ventral view; (**E**) MNG 16969 left femur in ventral view; (**F**) same as (**E**) in dorsal view; (**G**) MNG 16969 right tibia in ventral view; (**H**) same as (**G**) in dorsal view; (**I**) MNG 16314 proximal femur in ventral view; (**J**) MNG 14955 distal femur and associated shaft fragment in dorsal view; (**K**) MNG 17372 ulna in ventral view; (**L**) same as (**K**) in dorsal view; (**M**) MNG 11007 neural spine fragment; (**N**) MNG 10693 dorsal vertebrae and associated neural spines; (**O**) MNG 17439 neural spine fragment A; (**P**) MNG 17439 neural spine fragment B; (**Q**) MNG 17439 neural spine fragment C. For each element investigated histologically, the sampling location is indicated by a pink arrowhead.

This section includes the first bone paleohistological descriptions of *D. teutonis* long bone (Fig. 1A-L) and neural spine (Fig. 1M-Q) material recovered from the Bromacker locality of central Germany (Table 1). The bone microstructure of all comparative material of NA *Dimetrodon* species (Table 1) is described and illustrated in the Supplementary Information 1.

**Table 1:** List of German and NA *Dimetrodon* material investigated in the present study. CDI, cortico-diaphyseal index; Circ, circumference of the section; Cg, global compactness; CP, cortical porosity; CT, CT-scanned; dist., distal; D_max_, maximal diameter of the section; D_mean_, mean diameter of the section; L., left; N, no; prox., proximal; R., right; RBT, relative bone wall thickness; S, parameter S; T_mean_, mean cortical thickness of the section; TS, thin-sectioned; Y, yes; *, measurements and petrographic thin-sections processed for Shelton et al. (2012); ǂ, measurements and petrographic thin-sections processed for Knaus (2014).

Specimen number	Identification	Skeletal elements	Length (mm)	D_max_ (mm)	D_mean_ (mm)	T_mean_ (mm)	RBT (%)	CDI	Cg	S	Circ (mm)	CP (%)	TS	CT
Bromacker locality, Tambach Formation (upper Asselian), Thuringia, Germany														
MNG 10654	*D. teutonis*	L. humerus (midshaft)	103.29	9.96	8.37	0.65	7.77	0.134	0.37	0.035	29.7	-	Y	Y
MNG 10654	*D. teutonis*	L. tibia (midshaft)	87.65	7.65	6.65	0.47	7.07	-	-	-	-	-	Y	Y
MNG 10654	*D. teutonis*	L. fibula (midshaft)	90.11	5.81	4.52	0.43	9.51	-	-	-	-	-	Y	Y
MNG 16969	*D. teutonis*	L. femur (midshaft)	110.4	12.94	-	-	-	-	-	-	31.9	-	Y	Y
MNG 16969	*D. teutonis*	L. tibia (prox. shaft)	87.92	9.01	7.02	0.81	11.54	-	-	-	-	-	Y	Y
MNG 16969	*D. teutonis*	R. tibia (midshaft)	91.23		6.6	0.85	12.88	-	-	-	-	-	N	Y
MNG 17372	cf. *D. teutonis*	R. ulna (midshaft)	75.54	5.86	4.49	0.64	14.25	-	-	-	-	-	N	Y
MNG 14955	cf. *D. teutonis*	Femur (dist. shaft)	-	10.30	8.71	0.81	9.30	-	-	-	30.9	-	Y	N
MNG 16314	cf. *D. teutonis*	R. femur (prox. shaft)	88.7	10.80	9.71	0.88	9.06	-	-	-	-	-	Y	N
MNG 12675	*D. teutonis*	Neural spine fragment	-	4.94	-	-	-	-	-	-	-	-	Y	N
MNG 10693	*D. teutonis*	Neural spine fragment	326.00	4.83	-	-	-	-	-	-	-	-	Y	N
MNG 11007	*D. teutonis*	Neural spine fragment	-	3.20	-	-	-	-	-	-	-	-	Y	N
MNG 17439 A	*D. teutonis*	Neural spine fragment	-	-	-	-	-	-	-	-	-	-	Y	N
MNG 17439B	*D. teutonis*	Neural spine fragment	-	6.42	-	-	-	-	-	-	-	< 1	Y	N
MNG 17439 C	*D. teutonis*	Neural spine fragment	-	4.13	-	-	-	-	-	-	-	-	Y	N
Briar Creek Bonebed, upper Nocona Formation (upper Asselian/Sakmarian), Texas, USA														
IPBSH-13	*D. natalis*	L. humerus (midshaft)	58.00	8.82	7.8	1.53	19.62	-	-	-	28*	-	Y *	N
IPBSH-4	*D. natalis*	R. humerus (midshaft)	120.00	13.87	12.06	2.36	19.57	0.380	0.70	0.116	42*	-	Y *	N
IPBSH-19	*D. natalis*	R. femur (midshaft)	98.00	12.96	10.76	1.77	16.45	-	-	-	38*	-	Y *	N
IPBSH-2	*D. natalis*	R. femur (midshaft)	137.00	18.35	-	-	-	-	-	-	52*	-	Y *	N
MB.R.6827.1-3	cf. *D. natalis*	Neural spine fragment	-	3.92	-	-	-	-	-	-	-	1.5	Y	N
Crescent site, Garber/Hennessey Formations transition (upper Artinskian), Oklahoma, USA														
OMNH 15044	cf. *D. giganhomogenes*	R. Femur (midshaft)	81.00	13.45	11.31	1.84	16.27	-	-	-	38ǂ	-	Y ǂ	N
Sid McAdams locality, lower Vale formation (Artinskian), Texas, USA														
TMM 30966-201	cf. *D. giganhomogenes*	L. Femur (midshaft)	142.00	23.11	18.34	3.83	20.88	-	-	-	59ǂ	-	Y ǂ	N
TMM 30966-49	cf. *D. giganhomogenes*	R. Femur (midshaft)	221.00	38.03	32.08	5.84	18.20	-	-	-	103ǂ	-	Y ǂ	N
TMM 30966-291	*Dimetrodon* sp.	L. Femur (midshaft)	124.00	15.49	13.84	2.1	15.17	-	-	-	45ǂ	-	Y ǂ	N
Pond Creek, Garber Formation (Artinskian), Oklahoma, USA														
OMNH 15060	*Dimetrodon* sp.	Humerus (prox. shaft)	-	12.54	11.07	3.11	28.09	0.523	0.80	0.122	-	-	Y	N
OMHN 15055b	*D. grandis*	Humerus (dist. shaft)	-	10.84	8.81	1.56	17.71	-	-	-	-	-	Y	N
OMHN 15055a	*D. grandis*	Humerus (prox. shaft)	-	10.88	9.39	2.22	23.64	-	-	-	-	-	Y	N

### Long bone histology of *Dimetrodon teutonis*

**Fig. 2:**
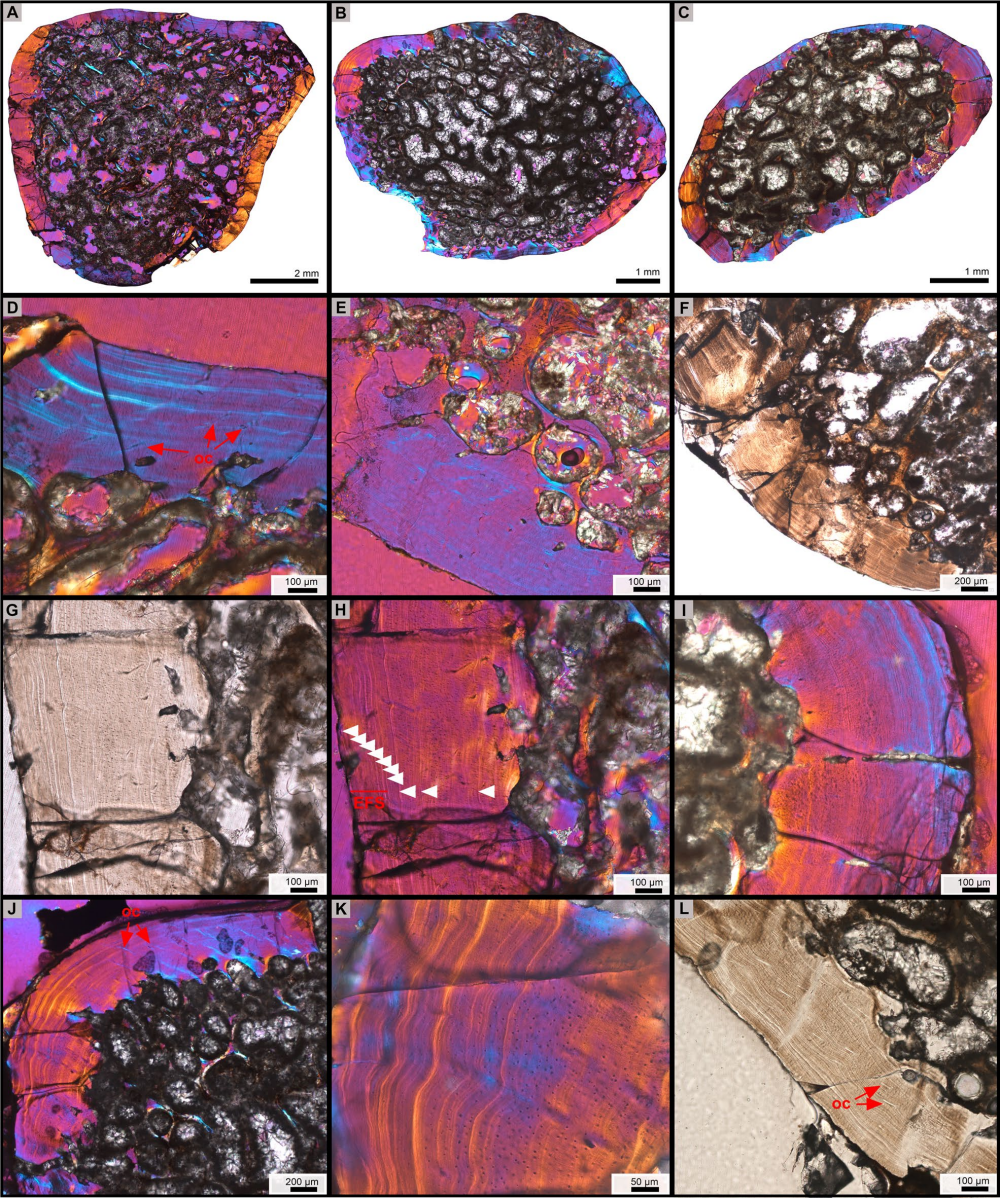
Long bone microstructure of *D. teutonis* MNG 10654. (**A**) mid-diaphyseal cross section of the left humerus; (**B**) mid-diaphyseal cross section of the left tibia; (**C**) mid-diaphyseal cross section of the left fibula; (**D**) Close up of the humeral cortex made of parallel-fibered tissue interrupted by several LAGs. Vascularization is low and mostly consists of simple and small oblique canals (as indicated by red arrows); (**E**) close up of the humeral cortex and perimedullary region showing secondary remodeled trabeculae. Remnants of an ontogenetically older periosteal tissue is visible in the core of some trabeculae; (**F**) close up on the poorly vascularized humeral cortex; (**G**,**H**) close up on the humeral bone wall interrupted by several LAGs (white arrowheads). The spacing between the LAGs decreases towards the periphery. An EFS is visible in the outer cortex; (**I**) close up on the poorly vascularized fibular cortex; (**J**,**K**) Close up on the tibial cortex interrupted by several growth marks. Oblique simple vascular canals are visible in some areas of the section (red arrows); (**L**) Close up of the tibial cortex showing the small and oblique simple vascular canals (red arrows). (**F**, **G**, **L**) in natural light; (**A**–**E**) and (**H**–**K**) in polarized light with lambda compensator. oc, oblique simple vascular canals.

All long bones investigated here present a comparable inner structure. The humerus, tibia and fibula of specimen MNG 10654 show similar microanatomy and histology at midshaft. The humerus (Fig. 2A) has a subtriangular cross-section, with a relatively thin compact bone wall (RBT = 7.8%; CDI = 0.134) and a relatively low global compactness (Cg = 0.37). The tibia and fibula (Fig. 2B, C) present more oval cross-sections with comparably thin cortices (RBT = 7-9.5%). In all three elements, the medullary region is filled with a loose trabecular network. The transition between the compact cortex and the medullary spongiosa is abrupt and well-defined (Fig. 2A-C), with a relatively low parameter S value (0.034) in the humerus. The cortex consists of a poorly vascularized parallel-fibered to lamellar tissue interrupted by LAGs (Fig. 2D-L). Few enlarged vascular canals can be found in the deeper cortex (Fig. 2D, G, H). The remaining vascularization consists of sparse, simple and oblique canals (Fig. 2D, J, L). In every long bone, the spacing between the LAGs decreases towards the periosteal surface and grades into an external fundamental system (EFS) (Fig. 2H, J, K), attesting this individual had reached skeletal maturity. Secondary osteons are absent in the cortex. Remodeling is restricted to the trabeculae and erosion bays at the perimedullary margin (Fig. 2E), and parts of the endosteal margin are still resorptive (Fig. 2D, G-I). Some trabecular cores exhibit remnants of an ontogenetically younger primary cortex that consisted of a somewhat more disorganized tissue with larger osteocyte lacunae (Fig. 2E).

**Fig. 3:**
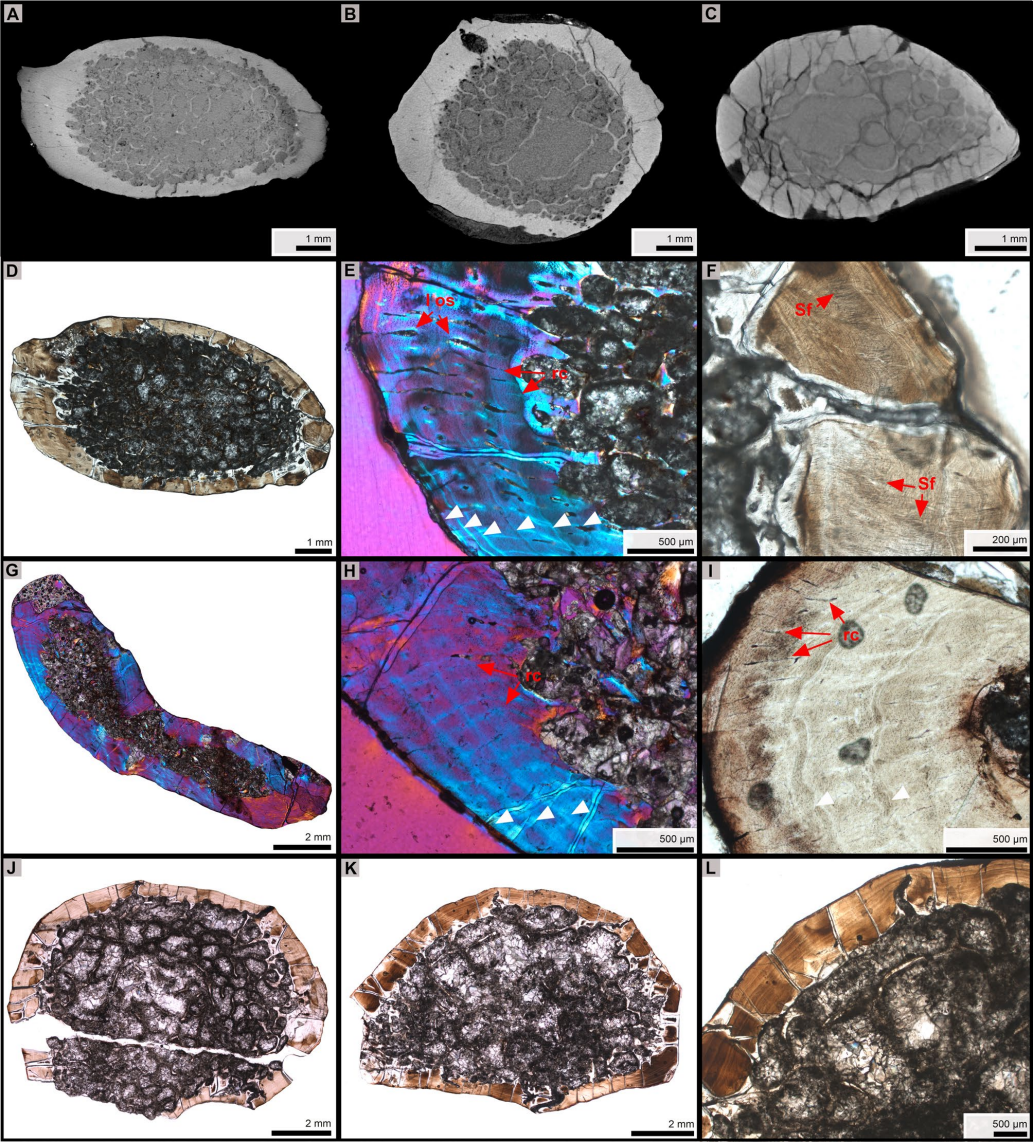
Long bone microstructure of selected *D. teutonis* specimens. (**A**) virtual proximal shaft cross-section of MNG 16969 left tibia; (**B**) virtual mid-diaphyseal cross-section of MNG 16969 right tibia. A nutrient foramen penetrating the cortex is visible at this level; (**C**) virtual mid-diaphyseal cross-section of MNG 17372 right ulna; (**D**) proximal shaft cross-section of MNG 16969 left tibia taken close to (**A**); (**E**) close up of MNG 16969 tibial cortex in (**D**). On this side of the section, the cortex is relatively well-vascularized, and consists in the alternance of zones and annuli associated with LAGs (white arrowheads). The onset of an EFS is visible close to the bone surface. Vascular canals consist of longitudinal or radial primary osteons (I os) as well as simple radial canals (rc); (**F**) close up on the tibial cortex of MNG 16969 left tibia. Note the presence of numerous bundles of Sharpey’s fibers (Sf) throughout the cortical thickness; (**G**) Mid-diaphyseal cross-section of MNG 16969 left femur. This element has been strongly crushed dorso-ventrally; (**H**) Close up on the femoral cortex of MNG 16969 interrupted by several growth marks (white arrowheads). Simple radial vascular canals (rc) are visible; (**I**) close up of the MNG 16969 femoral cortex showing the poorly vascularized cortex interrupted by growth marks (white arrow heads). Small simple radial canals (rc) are visible throughout the cortex; (**J**) Proximal shaft cross-section of MNG 16314 femur; (**K**) distal shaft cross-section of MNG 14955 femur; (**L**) close up on the poorly vascularized and parallel-fibered cortex of MNG 14955 femur. (**D**,**F**,**I**,**J**–**K**) in natural light; (**E**,**G**,**H**) in polarized light with lambda compensator.

The femur and tibiae of MNG 16969 belong to an individual slightly larger than MNG 10654 (fibular lengths of 93.3 and 90.1 mm, respectively). The tibiae have sub-circular to oval mid-diaphyseal cross-sections (Fig. 3A, B, D-F). Their cortical thickness varies around the sections, with RBT of 11.5–12.9%. The femoral midshaft (Fig. 3G-I) has been compressed dorso-ventrally during diagenesis. As a result, the trabecular network occupying the medullary region has collapsed and its RBT cannot be calculated. As in MNG 10654, all investigated elements present a medullary region filled by trabeculae and a well-defined transition with the compact cortex. The endosteal surface is resorptive and cortical remodeling is limited to a few erosion bays in the perimedullary region (Fig. 3E, F, H, I). Vascularization is relatively higher than in MNG 10654. The femur and sampled tibia possess a stratified cortex with three to four visible zones alternating with LAGs (Fig. 3E, H, I). The zones consist of vascularized parallel-fibered bone tissue with a few longitudinal to oblique primary osteons and/or more numerous simple radial canals (Fig. 3E, H, I), depending on the region of the section. Sharpey’s fibers are visible throughout the tibial cortex. No EFS could be observed in the femur. However, the tibia presents closely spaced LAGs near the periosteal surface that correspond to the onset of an EFS (Fig. 3E).

Two additional partial femora (MNG 16314 and 14955) assignable to *D. teutonis* were sampled close to midshaft and exhibit very similar microstructures (Fig. 3J-L). In both elements, the section is oval, the compact cortex is relatively thin (RBT about 9%), the endosteal margin is resorptive, and the transition with the medullary region is well-defined. The cortex is made of a poorly vascularized parallel-fibered bone tissue interrupted by LAGs (Fig. 3L). The vascularization consists mostly of sparse simple vascular canals with an oblique to radial orientation.

Finally, a right ulna (MNG 17372) also assignable to *D. teutonis* was CT-scanned. Its microanatomy matches all other investigated long bones (Fig. 3C). The oval mid-diaphyseal section presents a seemingly poorly vascularized compact cortex with a RBT of 14.3%. The medullary region is filled by a loose trabecular network.

### Neural spine histology of *Dimetrodon teutonis*

**Fig. 4:**
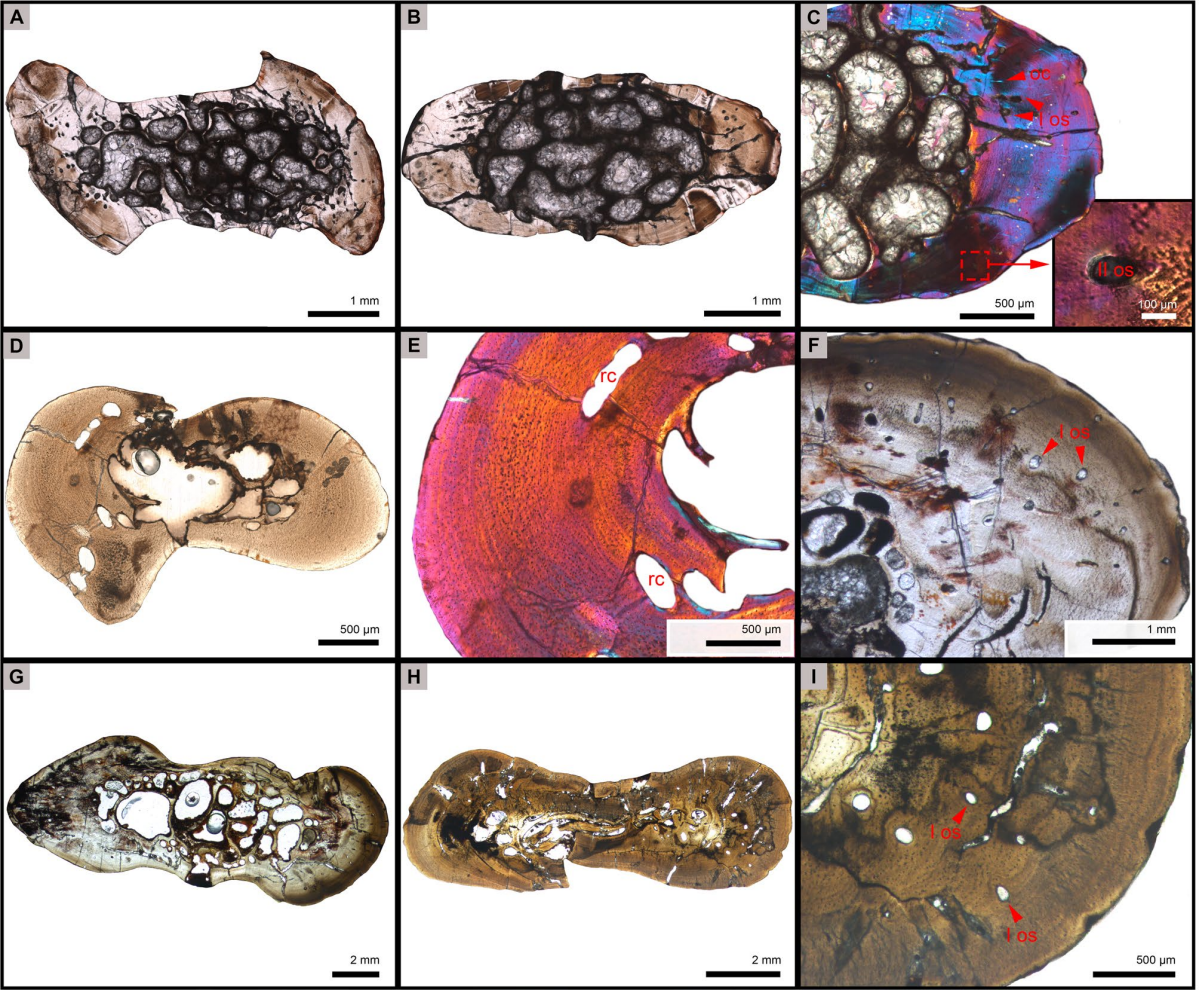
Neural spine microstructure of *Dimetrodon teutonis*. (**A**) proximal cross section of the largest neural spine of MNG 10693; (**B**) cross section of MNG 12675; (**C**) Close up on the poorly vascularized and parallel-fibered cortex of MNG 12675. Vascular canals consist of a few enlarged longitudinal primary osteons (I os), isolated incipient secondary osteons (II os), and small simple oblique canals (oc). (**D**) cross section of the small neural spine of MNG 11007; (**E**) close up on the nearly avascular cortex of MNG 12675. A few resorption cavities (rc) boarded by a thin layer of endosteal lamellar bone are visible in the deep cortex; (**F**) Close up on neural spine fragment A of MNG 17439. Again, the lateral cortex is poorly vascularized. Vascular canals mostly consist of longitudinal primary osteons (I os) of variable lumen size; (**G**) Cross section of neural spine fragment B of MNG 17439; (**H**) Cross section of neural spine fragment C of MNG 17439. The lateral cortices are poorly vascularized. Vascular canals are either longitudinal primary osteons or small simple radial canals; (**I**) close up of the lateral cortex in (**H**). Vascularization is low and consists of a few isolated longitudinal primary osteons (I os).

The cross-sectional shape of MNG 10693 is reminiscent of the characteristic dumbbell described in other NA *Dimetrodon*, but with a relatively wider medial groove (Fig. 4A). MNG 12675 presents a more oval shape (Fig. 4B). In both specimens, the lateral cortices are thicker than the medial ones and composed of a poorly vascularized parallel-fibered tissue regularly interrupted by LAGs (Fig. 4C). Most vascular canals consist of longitudinal primary osteons, some enlarged by erosion, located in the inner half of the cortex (Fig. 4C). Remodeling is restricted to a few incipient secondary osteons, as well as the deposition of thin layers of endosteal lamellar bone along the trabeculae and erosion bays bordering the medullary region (Fig. 4C). The medullary cavity is filled by a trabecular network (Fig. 4A-C). MNG 11007 consists of fragments of a smaller neural spine. The cross-section is also dumbbell-shaped (Fig. 4D). The lateral cortices are thick and made of a nearly avascular parallel-fibered bone tissue interrupted by poorly defined growth marks (Fig. 4D, E). A few large resorption cavities are present in the cortex, including some bordered by secondary lamellar bone tissue (Fig. 4D, E). The medullary cavity only shows a few trabeculae. Fragment A of MNG 17439 (Fig. 1O) consists in an incomplete cross-section preserving the lateral cortex made of parallel-fibered tissue interrupted by poorly defined growth marks (Fig. 4F). Again, vascular density is low with a few longitudinal primary osteons and simple vascular canals (Fig. 4F). Fragments B and C of MNG 17439 are similar in shape and histology (Fig. 4G-I) to MNG 10693 with lateral cortices made of poorly vascularized parallel-fibered bone tissue.

### Body mass estimates based on stylopodial circumferences

Following Campione & Evans^29^, the humeral and femoral midshaft circumferences of comparably-sized individuals (MNG 10654, MNG 16969 and MNG 14955) provide an estimated body mass of 6.3 to 6.8 kg for *D. teutonis*. Using the same method, the estimated body mass of fully grown *D. natalis* individuals (i.e. IPBSH-4 and IPBSH-2) is 20.9 to 21.7 kg.

## Discussion

### Histology and paleobiology of *Dimetrodon teutonis*

The bone histology of *D. teutonis* is investigated here for the first time, allowing a reassessment of previous taxonomic and ecological interpretations. The well-developed medullary region filled by secondary trabeculae and the presence of an EFS^22^ in the humerus, tibia, and fibula of MNG 10654, indicate that this individual was skeletally mature at death. These results support Berman et al.^2^ in identifying MNG 10654 as an adult despite its small size, and reaffirm *D. teutonis* as a distinct, diminutive species. Newly excavated material (MNG 16969) shows, however, that *D. teutonis* could attain slightly larger sizes.

All examined long bones exhibit uniform midshaft microanatomy. The relatively low global compactness and thin compact cortex (RBT between 7.1 and 14.2%) reflects sustained deep cortical resorption and medullary region expansion during ontogeny. Remnants of the ontogenetically older cortex are incorporated into secondary trabeculae occupying most of the medullary region, with an abrupt transition between cortex and spongiosa (i.e. low parameter S). Zeugopodial elements (ulna, tibia, fibula) tend to show thicker bone walls than stylopodials (humerus, femur), though our sample is limited.

At the histological level, the cortex consists primarily of poorly vascularized parallel-fibered to lamellar tissue interrupted by growth marks (annuli and/or LAGs). Age estimates remain uncertain because medullary cavity expansion has erased much of the growth record, but at least three to four annual cycles (excluding the EFS, whose temporal extent is unknown;^22^) are preserved in the thin cortices of humerus MNG 10,654 and the femur and tibia of MNG 16969. Vascularization is generally low, with occasional primary osteons and mostly small and oblique simple canals. Its density varies among individuals and may reflect ontogenetic stage or environmental conditions^22^. MNG 16969 exhibits higher vascularization in the femur and tibia (at least in some regions of the sections) than slightly smaller individuals (e.g. MNG 10654; MNG 16314). Despite its somewhat larger size, the absence of a clear EFS in its femur and the early development of one in its tibia suggest MNG 16969 may have been ontogenetically younger than MNG 10654. These observations suggest a degree of developmental plasticity in *D. teutonis.* Developmental plasticity or sexual dimorphism have likewise been invoked to explain age-size discrepancies in *D. natalis*^1,9^.

The lack of young juvenile long bone material currently limits the reconstruction of early growth dynamics in *D. teutonis*. Extensive medullary region expansion further complicates interpretations of early growth stages. However, the cortex is well preserved in neural spines. Both small (likely young individuals; e.g. MNG 11007) and larger neural spines show thick lateral cortices of poorly vascularized parallel-fibered to lamellar tissue (CP < 1%), indicating slow growth throughout much of ontogeny in these axial elements. Overall, osteohistological data point to a slow growth rate in *D. teutonis*, consistent with its small adult size.

### Histology and paleobiology of NA *Dimetrodon* species

The long bone histology of NA *Dimetrodon* species has been previously described, though often from fragmentary or poorly identified material (e.g.^25^). The most comprehensive work remains that of Shelton et al.^9^, who analyzed humeral and femoral ontogenetic series of *D. natalis* from Briar Creek, Texas.

The material newly analyzed and reinvestigated here (Supplementary Information 1) consists mainly of stylopodial elements from different-sized *Dimetrodon* species (*D. natalis*, *D. grandis*, and cf. *D. giganhomogenes*) from four lower Permian lowland localities of Texas and Oklahoma. *D. natalis*, the smallest known NA species, reached an adult body mass of 21–37 kg, whereas *D. giganhomogenes* and *D. grandis* exceeded 166 kg and 200 kg, respectively [1, 2, 30, present study]. Despite this substantial body size disparity, our observations corroborate Ricqlès’^25^ conclusions that lowland NA *Dimetrodon* species share a remarkably conservative bone microstructure.

Long bones consistently show high global compactness values and thick compact cortices (RBT 15.2–28.1%). Periosteal bone consists of well- to highly-vascularized woven or parallel-fibered tissue interrupted by thin annuli and/or LAGs. Vascularization has a predominant radial organization, with longitudinal primary osteons arranged in radial rows and frequently interconnected by radial anastomoses. Simple radial canals are also common. These vascular features indicate relatively rapid growth dynamics during most of ontogeny in these taxa^9,22^. Remodeling remains limited to the endosteal margin and the secondary trabeculae of the medullary region, even in adults. Contrary to Ricqlès’^25^ observations, our results show the cortex-medullary region transition is not always conspicuous and abrupt. In juveniles (e.g., *D. natalis* IPBSH-13, *D. grandis* OMHN 15055) this transition is poorly defined due to progressive resorption of the deep cortex. Adults often show a sharper boundary (e.g. IPBSH-4), though some fully grown specimens with an EFS retain a gradual transition (cf. *D. giganhomogenes* TMM 30966-49; *Dimetrodon* sp. OMNH 15060).

In *D. natalis*, the compact cortex remains both thick (RBT > 16%) and well-vascularized throughout ontogeny (Table 1;^9^). Shelton et al.^9^ estimated skeletal maturity at a maximum age of 11–17 years based on retrocalculated growth cycles(^9^: Table 1), but our reexamination of the material suggests this estimate may be too high. Their method for inferring missing growth cycles - dividing the distance between the medullary cavity center and the deepest visible LAG by the largest interval between adjacent LAGs - implicitly assumes that each annual cycle is recorded by a LAG. Yet, annual growth cycles in *D. natalis* may comprise one annulus and one zone, as recognized by both^9^ and the present study. The thickness of bone deposited per annual cycle varies in *Dimetrodon*^25^, but generally decreases with ontogeny, as in other tetrapods^22^. Consequently, early cycles should be the thickest. For example, humerus IPBSH-13 exhibits a single LAG directly beneath the periosteal surface, indicating the entire cortex represents a single (likely incomplete) growth cycle. Yet, at this stage, the mid-diaphyseal circumference already reached 66% of the largest known *D. natalis* humerus (IPBSH-4). Moreover, because long bones begin forming before hatching, the medullary region center does not mark the onset of post-hatching growth. Taken together, the predominance of well-vascularized woven-parallel complex deposited through most ontogeny suggests *D. natalis* grew relatively rapidly and attained skeletal maturity over a probably shorter time period than estimated by^9^. Conducting a skeletochronological reassessment in *D. natalis* to get a more accurate age estimate at skeletal maturity is, however, beyond the scope of this paper.

The hyperelongated neural spine of the diminutive *D. natalis* (MB.R.6827.1-3) from Briar Creek displays less-vascularized cortices (CP 1.5%) than appendicular elements. This contrasts with medium to large-bodied species, whose neural spines show highly vascularized cortices reflecting rapid distal outgrowth^23,25,26^. Indeed, neural spine cortical porosity in *D. grandis* and *D. giganhomogenes* is 13.5–16%^26^. Our preliminary observations therefore suggest differential growth rates between the dorsal sail and the rest of the skeleton in *D. natalis*; a hypothesis that should be further tested with additional material. Relatively slower neural spine growth may characterize diminutive species and aligns with reports that small species had proportionally smaller sails than larger forms^2,12,30^. Likewise, Brink et al.^4^ reported on a small, yet undescribed, *Dimetrodon* species from Richards Spur (Oklahoma) with poorly-vascularized neural spine cortices (CP = 6.3%; ROM 73637). Comparable observations are reported here for *D. teutonis* neural spines.

Additionally, our new data document a third case of sympatric *Dimetrodon* species in a lowland NA ecosystem. Previous work^9,10^ has shown diminutive species coexisting with larger congeners. Shelton et al.^9^ found *D. natalis* living alongside a much larger congeneric taxon (*D. booneorum* and/or *D. limbatus*) and the sphenacodontid *Secodontosaurus*^1^ in the late Asselian/early Sakmarian Briar Creek fauna. Similarly, Knaus^10^ identified co-occurring species at the Artinskian Sid McAdams locality, Texas: the large *D. giganhomogenes* (TMM 30966-201, TMM 30966-49) and a smaller unnamed species (TMM 30966-291) with femoral size and histology comparable to *D. natalis*. In our study, the histology, size and morphology of humerus OMNH 15060 indicate that it belonged to a skeletally mature individual of a diminutive *Dimetrodon* species coexisting with the much larger *D. grandis* at the Artinskian Pond Creek locality, Oklahoma. OMNH 15060 preserves at least five growth cycles before EFS formation and shows limited medullary cavity expansion. This suggests this animal reached adult size in just over five years and grew rapidly during favorable seasons, as indicated by radial vascularization within the zones^22^. Its shaft circumference approximates that of adult *D. natalis* humerus (IPBSH-4). The well-developed proximal articular surface confirms skeletal maturity^8^. In contrast, the similarly-sized *D. grandis* humerus OMHN 15055 shows continued growth through most of the cortex (a single LAG visible near the periosteal surface) and a less-developed proximal articular surface, implying the juvenile stage of a larger species and confirming the co-occurrence of small and large-bodied *Dimetrodon* species also at Pond Creek.

### Environmental constraints on the life history and ecology of diminutive *Dimetrodon* species

Our study first shows that NA and German diminutive *Dimetrodon* species had drastically different long bone microanatomies. This is best exemplified by the humeral global compactness values of *D. natalis* adults and OMNH 15060 that are about twice as high as that of *D. teutonis* MNG 10654, due to thicker compact cortices and a denser medullary spongiosa. Long bone microanatomy is known to be indicative of lifestyle habits (e.g. aquatic, amphibious, terrestrial, see review in^22^), with amphibious taxa generally showing thicker bone walls and more medullary trabeculae than their strictly terrestrial, similarly-sized relatives^22^. While *D. teutonis* inhabited a strictly terrestrial environment^7^, an amphibious lifestyle, or at least a diet based on aquatic taxa, has been proposed for some NA *Dimetrodon* species that inhabited lowland environments with aquatic-based food chains^11,31^. The relatively high compactness values observed in the long bones of *D. natalis* and OMNH 15060 could thus be in support of a somewhat amphibious lifestyle. In such a scenario, diminutive *Dimetrodon* species could have favored small aquatic or amphibious preys, while larger sympatric species would have fed on more terrestrial taxa. This hypothesis needs to be tested in the future by investigating other lines of evidence (e.g., coprolite contents or isotopic composition of *Dimetrodon* remains), which is, however, beyond the scope of the current study.

**Fig. 5:**
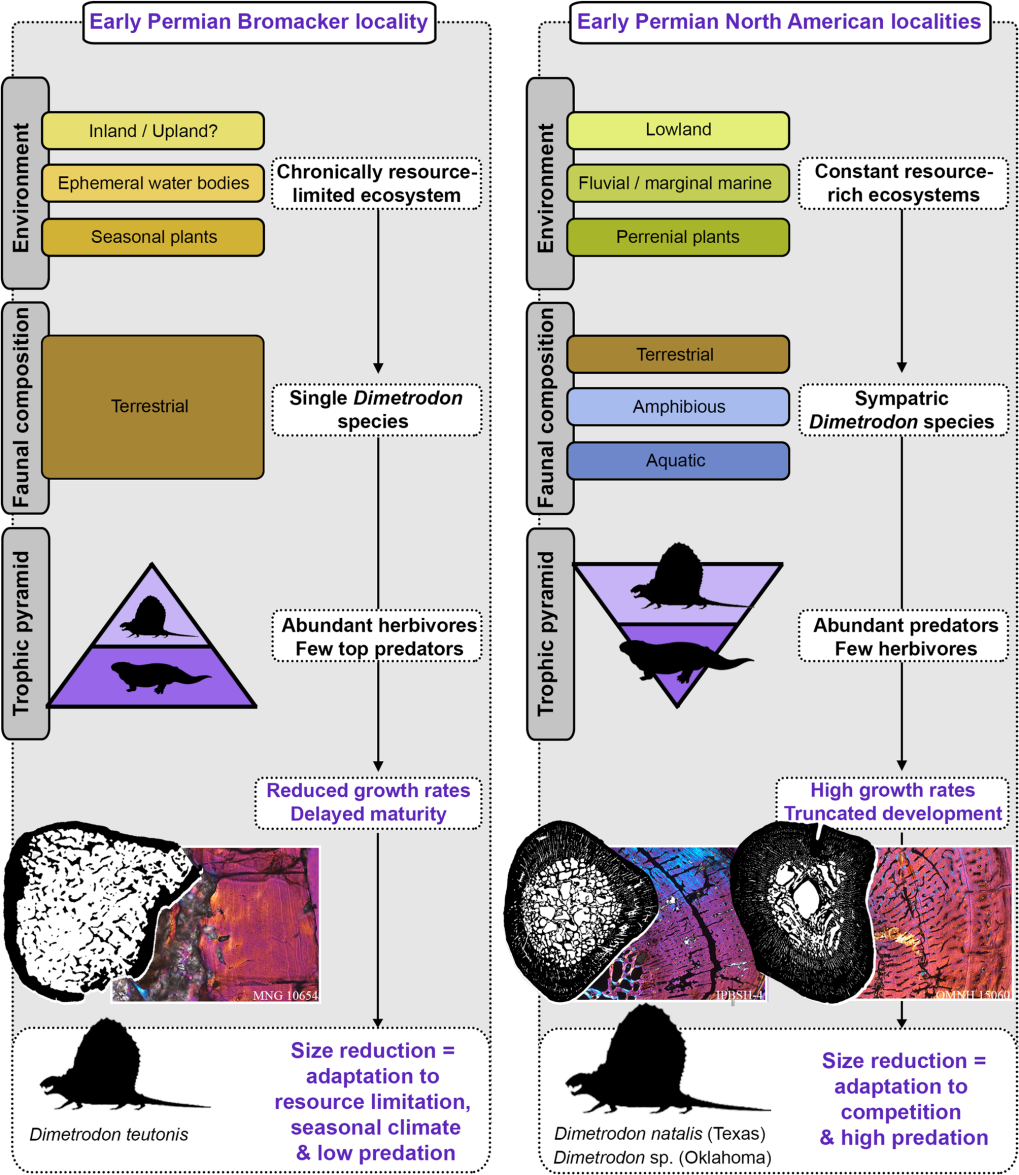
Environmental effects on the growth strategies of diminutive *Dimetrodon* species. This figure summarizes the differences in terms of environmental conditions, faunal composition, and trophic pyramid structure between the lower Permian Bromacker locality of central Germany and most coeval NA localities. We propose that these differences explain the different growth strategies employed by the diminutive *Dimetrodon* species that inhabited these ecosystems.

More importantly, our osteohistological investigation also reveals that the diminutive *Dimetrodon* species from North America and Germany reached reduced body sizes through fundamentally different developmental strategies (Fig. 5). *D. natalis* most likely achieved its small body size through truncated development, but kept relatively fast growth rates throughout most of its ontogeny, a character likely plesiomorphic for all *Dimetrodon* species. Indeed, despite subtle interspecific variability, all NA species investigated histologically exhibit well- to highly-vascularized long bone cortices with a preferential radial vascular organization [9, 10, 25, present study]. In contrast, *D. teutonis* exhibits reduced growth rates, probably over an extended period of time (see below). We therefore propose that paleoenvironmental factors, rather than phylogenetic constraints, played a role in shaping the life history strategies of these species; a hypothesis already put forth by Berman et al.^2,12^ for *D. teutonis*.

Adaptive shifts in body size driven by environmental constraints have been extensively documented in vertebrates, in particular with regards to cases of gigantism and dwarfism in insular ecosystems (e.g.^32^). Models such as Palkovacs’^33^ provide a framework for understanding how selective pressures on islands shape the life-history strategies of organisms. Life-history traits (e.g., size and age at maturity, longevity) are organized along a “fast–slow continuum,” with small adult size, early maturity, and short lifespan at the fast end, and the opposite strategy, i.e. large adult size coupled to delayed maturity and an extended lifespan at the slow end^34^. Factors including temperature, seasonality^32^ but more importantly extrinsic mortality related to predation pressure, and resource availability, influence growth rate and age at maturity, which in turn determine adult size^33^. For instance, the combination of resource limitation and low extrinsic mortality typical of insular ecosystems often leads to a marked reduction in adult body size - or “insular dwarfism” - in vertebrates otherwise large on the mainland. This reduction results from a deceleration in individual growth rates coupled with an increased age at maturity, as animals invest more time and energy in resource acquisition^33,35^. Low predation further relaxes selective pressures for rapid juvenile growth, allowing for smaller body sizes to thrive^36^.

Although growth rate deceleration leading to size reduction is well documented in insular species^34,36–38^, similar processes in continental settings remain underexplored. A notable exception is the study by Orlandi-Oliveras et al.^21^, who investigated the life history traits of two dwarfed hipparionin lineages from mainland Greece and Spain during the Miocene. Interestingly, they found that both dwarf species achieved small body size through contrasting growth strategies. Using paleohistological and paleoenvironmental data, they inferred that resource limitation led to growth-rate deceleration and delayed maturity in the Spanish taxon. To the contrary, strong predation pressure favored truncated development and early maturity in the dwarfed Greek form.

Another relevant example is provided by Hyeun-Ji et al.^39^, who investigated dwarfism in geographically connected continental populations of extant amphibians. Despite the lack of genetic isolation, dwarf populations that experienced drier, warmer microclimates, had distinct trophic statuses, and exhibited lower growth rates than their non-dwarf relatives^39^. This case evokes the concept of inselbergs — isolated continental microhabitats, or “terrestrial islands” — that support unique biological communities and experience evolutionary dynamics that can be comparable to true islands^40^. Inselbergs are typically associated with harsh environmental conditions, to which resident organisms evolve specialized ecological adaptations^40^.

Several lines of evidence suggest that the Bromacker ecosystem might represent an inselberg analogue, providing a compelling explanation for the unique growth pattern of *D. teutonis*. The Tambach Basin, which includes the Bromacker locality, has been interpreted as a small inland, internally drained, and geographically isolated paleograben^16^. Multiple indicators also point to chronically limited resources and a highly seasonal climate in the lower Permian Tambach Formation. Estimated mean annual paleotemperatures of 10.9–15.0 °C suggest that Bromacker was relatively cool for a subequatorial continental locality, and probably at some elevation^16,18^. In addition, the frequent occurrence of large desiccation cracks and contour marks produced by water evaporation indicate prolonged dry periods (^16,17^; Fig. S1A, B). Aquatic or semi-aquatic tetrapods were also absent in this ecosystem and water bodies were ephemeral^7,41^.

Different small to medium-sized tetrapod species might have been fossorial at Bromacker, as attested by the presence of different-sized vertebrate burrows and associated scratch traces (^42–44^; Fig. S1A, C, D, F). Morphological features of the skull and limbs of the recumbirostran “microsaurs” recovered from the Tambach Formation are in agreement with a fossorial lifestyle (^45,46^; Fig. S1G). Moreover, some specimens of diadectids and of the caseid *Martensius bromackerensis* were found articulated within burrow infills^44^. This further suggests that such species might have used burrows during life, for estivation or shelter during dry periods, for example^43^. The use of burrows by different potential prey species in the Bromacker ecosystem made these animals likely difficult to reach, especially for *D. teutonis* whose backsail prevented it from entering even the largest burrows^12^.

The herbivorous diadectids were the most speciose and abundant tetrapods at Bromacker^7^. The heavily-trampled surfaces of diadectid footprints found at the same locality (e.g.^47)^, might suggest a potential gregarious behavior for these animals^48^, making them, again, more difficult to prey upon. All these lines of evidence suggest somewhat limited resources for *D. teutonis*, which were even more scarce during the dry season.

Reduced predation pressure and competition may also have favored small size in *D. teutonis*. The only other apex predator present in the Bromacker ecosystem—the varanopid *Tambacarnifex unguifalcatus*—had a comparable estimated snout–vent length (50–60 cm) to *D. teutonis*^49^.

In summary, our osteohistological study shows that *D. teutonis* reached its diminutive body size through decelerated growth rates (Fig. 5) compared to the expected plesiomorphic condition in this genus. Reduced growth rates were probably associated with a delayed age at maturity, as predicted by life history models (e.g.^33,35^). The intraspecific variability in vascularization density observed in our small *D. teutonis* sample might reflect physiological plasticity to adapt to fluctuating resources, as seen in extant ectotherm vertebrates^35^. Chronically limited resources and low predation pressure in the unique Bromacker ecosystem likely constrained this predator’s life history (Fig. 5).

By contrast, despite their relatively small body sizes, *D. natalis* and specimen OMNH 15060 exhibited rapid, though episodic, growth throughout development (Fig. 5). This is evidenced by the presence of highly-vascularized woven to parallel-fibered bone tissue with predominantly radial vascular organization (^9^; present study). Consequently, these species likely reached skeletal maturity within only a few years^33,35^; probably just over five years in the case of OMNH 15060.

In NA lowland localities, several diminutive *Dimetrodon* species coexisted with much larger congeners [9, 10, present study], as well as other large and common predators, e.g., the large sphenacodontid *Secodontosaurus* at the Briar Creek locality^4^. Even though these environments were rich in resources — featuring abundant aquatic and terrestrial prey, permanent water bodies, and perennial vegetation^5,6^— interspecific competition within the predator guild may have driven body-size differentiation in some *Dimetrodon* species, promoting niche partitioning and reducing interspecific competition^21,35^. Moreover, the smaller *Dimetrodon* species in the NA lowland assemblages were likely more vulnerable to predation than *D. teutonis* from the Bromacker locality, given the presence of much larger coexisting carnivores in the lowlands. Rapid attainment of adult size would have conferred a significant advantage, enabling earlier reproduction and thus enhancing reproductive success^21,35^.

Interestingly, some NA diminutive *Dimetrodon* species were recovered from the Garber (OMNH 15060 in the present study) and Vale Formations (see^10,50)^, which are geologically younger and represent different climatic conditions than the upper Asselian - lower Sakmarian Briar Creek locality. During the Artinskian, intense CO₂-driven global warming led to increased aridity and seasonality at low latitudes, marking the transition from the late Paleozoic Ice Age to greenhouse conditions^51,52^. Nevertheless, this climatic shift does not appear to have markedly influenced the life history strategies of the small *Dimetrodon* species. This further supports the interpretation that competition and predation, rather than climate or resource availability, played the primary role in shaping the growth patterns of NA diminutive *Dimetrodon* species (Fig. 5).

## Conclusions

Our results show that the diminutive *Dimetrodon* species from North America and Germany had fundamentally different bone microanatomy and life history strategies to reach their dwarf size. *D. teutonis* achieved its small size through slow growth and possibly delayed maturity, likely as an adaptation to resource limitation, seasonal climate, and low predation pressure in the unique early Permian Bromacker ecosystem. In contrast, osteohistological and paleoenvironmental evidence indicate that NA diminutive species grew more rapidly but reached maturity early, probably in response to interspecific resource competition and strong predation pressure in early Permian lowland ecosystems. These contrasting growth strategies reflect differing ecological pressures and illustrate multiple evolutionary pathways to dwarfism in this iconic genus.

## Material & methods

### Fossil material and its provenance

#### *Dimetrodon teutonis* and associated ecosystem

All *D. teutonis* specimens yet discovered come from the lower Permian Bromacker locality in the Tambach Formation of central Germany^2,12^. The age of the locality is late Asselian, between 295.8 ± 0.4 Ma and 294.1 ± 0.4 Ma, based on radioisotopic ages of the upper part of the Tambach Formation and of the underlying Rotterode Formation and biostratigraphical correlations^15,53,54^. The Bromacker locality is known for its exceptional vertebrate skeletal and trace fossil record (Fig. S1), as well as invertebrate traces, arthropods, and rather rare plant remains (^7,41,43,44,55^; Fig. S1). This paleoecosystem was unique and differed from most coeval fossil assemblages, mostly in North America, despite all being close to the paleoequator at the time^7^. Its tetrapod fauna, consisting of relatively small temnospondyls, seymouriamorphs, recumbirostran ‘microsaurs’, captorhinomorphs, and bolosaurids, as well as medium-sized diadectids and early synapsids, was exclusively terrestrial with abundant herbivores and few predators^7^. This locality belongs to the Bromacker-Sandstein Member, and is characterized by fluvial to floodplain deposits (^53,56^; Fig. S1A). The bone layers are preserved in a 1 m thick fine-grained cross-stratified, thinning-upwards sandstone interval with an erosive base, possibly crevasse splay deposits within a 4 m-thick sequence mostly composed of laminated mudstone, probably levee to overbank deposits (Fig. S1A). Tetrapod footprints are preserved mostly in the underlying 7 m of tabular medium-grained sandstones, probably channel deposits (Fig. S1A, B). Both these sandstone deposits are also characterized by tetrapod burrows and scratch traces (^43^; Fig. S1D, C, F). The Bromacker locality was an inland, probably internally-drained isolated basin and has been hypothesized to be at a relatively high altitude^16,18^.

The available *D. teutonis* material is limited and mostly consists of isolated bones and a few semi-articulated appendicular elements and vertebrae with elongated neural spines showing the characteristic dumbbell-shape in cross-section^2,12^. In the present study, we investigate the microstructure of the left humerus (Fig. 1A, B), tibia and fibula (Fig. 1C) of the referred specimen MNG 10654 (Table 1) described in^2^. These authors hypothesized that these bones belonged to a somatically mature individual based on the complete suture closure of its scapulocoracoid bones, the fully ossified tarsal elements, and the well-developed articular facets of its limb bones. We also sampled the left femur (Fig. 1D-F) and associated right tibia (Fig. 1D) of a newly excavated specimen MNG 16969 consisting of two partially articulated hindlimbs clearly assignable to *D. teutonis*. The associated left tibia (Fig. 1G, H) was only CT scanned. Comparison of MNG 10654 and MNG 16969 tibial and fibular lengths indicates that the latter specimen was only slightly larger than the former one (see Table 1). MNG 16969 is thus expected to have belonged to a fully-grown individual, if Berman et al. ‘s^2^ hypothesis holds true. Additionally, we documented the histology of two partial femora (cf. *Dimetrodon teutonis*, MNG 16314 (Fig. 1I) and MNG 14955 (Fig. 1J)), smaller but of comparable gross-morphology than the femora of MNG 16969, as well as an isolated right ulna (cf. *Dimetrodon teutonis*, MNG 17372, Fig. 1K, L). Finally, we also described the histology of hyperelongated neural spines sampled at different levels along their shafts, including specimens MNG 11007 (Fig. 1M), MNG 10693 (Fig. 1N), MNG 12675 (not figured) and MNG 17439 (Fig. 1O-Q). The referred specimen MNG 10693 comprises the longest and most complete neural spines ever documented for *D. teutonis*^2^. These neural spines belonged to an individual slightly larger than the *D. teutonis* holotype (MNG 10598) hypothesized by Berman et al.^12^ as a somatically mature individual based on the comparison of its presacral vertebral morphology to that of a juvenile *Dimetrodon* specimen from Texas^12^. We sampled the neural spine of a dorsal vertebra of MNG 10693 proximally (Fig. 1N). MNG 17439 is a newly uncovered specimen (2025 excavation) comprising dozens of isolated neural spine pieces. We sampled three different fragments presenting different sizes and morphologies (Fig. 1O-Q) and most likely coming from different portions of the sail. Permission was obtained from the Stiftung Friedenstein Gotha, Germany, for fossil access and destructive sampling. No ethical approval was required to study these specimens.

#### Comparative *Dimetrodon* material & associated ecosystem

In order to compare the histology of *D. teutonis* with its NA relatives, we reinvestigated previously made thin-sections, but also processed new slides of NA *Dimetrodon* species recovered from different localities, including both marginal marine and more inland paleoenvironments (Table 1). Permissions for fossil access and destructive sampling were obtained from the relevant institutions and no ethical approval was required to study these specimens.

The bone microstructure of all these NA *Dimetrodon* specimens is described and illustrated (Figs. S2 and S3) in Supplementary Information 1.

This material comprises the humerus (IPBSH-13) and femur (IPBSH-19) of two juvenile *Dimetrodon natalis* individuals from the Briar Creek locality of Texas, 48 and 72% respectively of the adult length documented for this species, as well as a fully-grown humerus (IPBSH-4) and femur (IPBSH-2) (see^9^). We also sampled, for the first time, an hyperelongated neural spine from the same locality referrable to *D. natalis* (MB.R.6827.1-3) based on morphology and size. The Briar Creek Bonebed is a 30 cm-thick gray mudstone, interpreted as a freshwater swamp or oxbow lake in a floodplain depositional setting^57^. Bones there are mostly disarticulated and were subjected to partial decay before burial. The vertebrate assemblage includes aquatic diplocaulids and anthracosaurs, aquatic and terrestrial temnospondyls, a diadectid, a bolosaurid and different synapsids, such as *Edaphosaurus*, *Ophiacodon* and *Dimetrodon*^57^. *Dimetrodon* is represented by at least two species of different sizes^9^. This locality is part of the Nocona Formation, which can be correlated with the Coleman Junction and the Elm Creek Formations, dated as upper Asselian to lower Sakmarian with conodonts^58^.

We also looked at the femoral histology of three individuals of the larger *Dimetrodon giganhomogenes* that supposedly died at different ontogenetic stages (OMNH 15044, TMM 30966-201, TMM 30966-49), as well as one individual (TMM 30966-291) of a sympatric and potentially diminutive *Dimetrodon* species^10^. Sections of these specimens were processed in the framework of the Bachelor’s thesis of one of us^10^. Specimen OMNH 15044 was recovered from the Crescent site of the Garber/Hennessey Formations transition zone of Oklahoma, where the most common tetrapods are the large captorhinid *Labidosaurikos* and *Dimetrodon giganhomogenes*^57^. This transition zone is probably of upper Artinskian age, being higher in the section than the Richards Spur locality, which has been dated with radiogenic isotopes to 289 − 286 Ma^14^.

Specimens TMM 30966-201, TMM 30966-49 and TMM 30966-291 come from the Sid McAdams locality of the Vale Formation of Texas. At this locality, freshwater fishes and amphibians, such as *Trimerorhachis* and *Seymouria* are fairly common, but *Dimetrodon giganhomogenes* is the most abundant terrestrial tetrapod^50^. The Vale Formation is a marginal marine unit characterized by prevalent fine-grained overbank floodplain deposition^50^ Its age can be considered approximately as upper Artinskian by biostratigraphy and correlations with Oklahoma stratigraphic units^14,52^.

Finally, we processed new petrographic sections from three humeral fragments from the Pond Creek locality of the Garber Formation of Oklahoma, USA (Table 1). These include a proximal humerus (a) and a distal humerus (b) bearing the same catalog number (OMNH 15055). It is not mentioned in the collection records whether they belonged to a single individual. However, they are both listed as *Dimetrodon grandis* juveniles. The third fragment (OMNH 15060) consists of a proximal humerus of an unidentified *Dimetrodon* species. The Pond Creek locality has been reasonably studied and holds aquatic, amphibious and terrestrial vertebrates, including freshwater sharks and osteichthyans, several amphibians such as *Eryops* and *Diplocaulus*, but also a *Diadectes* sp., and probably two different-sized *Dimetrodon* species, including *Dimetrodon grandis*^59^. The Garber Formation was not intensively studied from a sedimentological and depositional environment perspective. Kenney^60^ and Callahan^61^ proposed a floodplain environment with meandering rivers and evidence of aridity such as vertisol and mud cracks, and they rejected the hypothesis of deltaic and marginal marine environments (e.g.^62^). The Garber Formation is likely of middle to upper Artinskian age (e.g.^14,52^).

#### *Notes on the diminutive nature of some* Dimetrodon *species*

Although the phylogenetic affinities within Sphenacodontidae, and particularly the genus *Dimetrodon*, are poorly resolved^3,63^, the small body size observed in the diminutive species of this group is unlikely to represent a plesiomorphic condition. Whereas *D. natalis* has been regarded as a basal member of the genus by several authors^1,12^, the phylogenetic position of *D. teutonis* remains uncertain, and comprehensive taxonomic and phylogenetic reanalyses of this genus are needed^3^. Although species of *Dimetrodon* exhibit a general trend of body size increase throughout the early Permian^63^, parsimony suggests that the earliest members of the genus were plesiomorphically larger than *D. natalis* and *D. teutonis*, given the relatively large size of most other sphenacodontids^1,63^. Indeed, closely related taxa such as *Secodontosaurus*, *Ctenospondylus*, *Ctenorhachis*, or *Sphenacodon* have inferred adult body masses ranging from 52 to 138 kg^63^. Consequently, the small adult body size (< 37 kg;^2^) of these diminutive *Dimetrodon* species most likely represents a secondary size reduction and should be considered autapomorphic^2,12^.

### Steps taken before destructive sampling

To preserve a record of their 3D gross morphology and microanatomy, some complete and/or figured long bones recovered from the Bromacker locality (Table 1) were micro-CT scanned before destructive sampling at the Museum für Naturkunde, Berlin, using a FF85 dual-tube system (YXLON International, Hamburg, Comet Group). These bones were first scanned in their entirety with a 190 kV multifocus tube in microfocus mode. Higher-magnification scans of the mid-shafts were also made using the nanofocus mode. All virtual sections were then obtained and analyzed in Amira 3D 2023.2 (ThermoFisher Scientific). The different skeletal elements were also photographed and measured using a digital caliper (Table 1). In some cases, the mid-diaphyseal region to be sampled was molded using a dental silicone (Vinylpolysiloxane precision impression material) from Provil novo (Putty soft regular set) that cured in about three minutes and could then be carefully removed from the specimen.

### Destructive sampling and bone repair

A roughly 0.5 cm-thick slice of bone was taken from the shaft of the different skeletal elements using a NSK Ultimate XL Micromotor equipped with JOKE diamond tools and specialty diamond blades (9-S1031 by Botzian & Kirch). Whenever possible, limb bone samples were taken close to the growth center (which is around mid-diaphysis in these elements) because it corresponds to the portion of the shaft where the cortex is the thickest and growth record is maximal^22^. The sampled portions were reconstructed using a kneadable epoxy (Aves APOXIE Sculpt Modeling compound) using the silicone molds that were made before destructive sampling.

### Thin-sectioning protocol and image acquisition

We processed ground-sections of some of the specimens studied (Table 1) following standard petrographic protocols^64^ at the Friedenstein Stiftung Gotha, Gotha, Germany. The extracted bone samples were embedded in EPO-TEK^®^ 301-1 resin (Epoxy Technology Inc). For each skeletal element, two or more wafers of embedded bone were then cut using a Buehler IsoMet Low Speed Saw equipped with a Buehler IsoMet Wafering Blade (Series 15HC; No. 11-4245), affixed to frosted petrographic glass slides with epoxy, and finally ground to desired thickness (100–80 μm) with a Buehler EcoMet 30 polisher and Buehler CarbiMet SiC abrasive papers with decreasing grit sizes (P240, P400, P800, P1200). Ground sections were examined and photographed using a Zeiss Axioscope 7 microscope equipped with an Axiocam 305 color camera. Histological images were captured under normal and cross-polarized (with or without a lambda compensator) light using the software ZEN Core v3.3.

### Bone microstructural descriptions

All bone paleohistological terminology used in the present study follows^22^. In order to compare the bone microanatomy of *D. teutonis* with that of its NA relatives, different parameters were extracted (mainly from the stylopods and hyperelongated neural spines) and recorded in Table 1. Unless stated otherwise, most of these parameters were directly measured from scaled images in Adobe Photoshop CS6 or ImageJ and include: (1) the maximal width of the section at midshaft (D_max_); (2) the mean diameter of the section (D_mean_) that corresponds to the average of at least six diameters measured around the section; (3) the mean cortical thickness (T_mean_) of the section that corresponds to the average of at least a dozen measurements of bone wall thickness taken along the previously recorded diameters; (4) the relative bone wall thickness (RBT) expressed as a percentage and calculated as the ratio between T_mean_ and D_mean_. A low RBT value corresponds to a relatively thin cortex, whereas higher RBT values indicate thicker cortices (see^65^); (5) the cortico-diaphyseal index (CDI), which corresponds to the thickness of the cortex divided by the radius of the bone, was calculated for three humeral cross-sections (MNG 10654, IPBSH-4, and OMNH 15060) in Bone Profiler^66^ after converting these sections into binary images (black for bone and white for voids, i.e. resorption cavities, medullary cavity; see Fig. 5). When the cortical thickness varies within the section, CDI calculations obtained in Bone Profiler are more accurate than RBT calculations. For a given section, RBT should approximate CDI/2; (6) the global compactness (Cg) of these three sections, as well as (7) the width of the transition zone between the compact cortex and the medullary cavity (parameter S) were also obtained from Bone Profiler^66^. S tends to be high in the presence of trabeculae between the free medullary cavity and the compact cortex and low when trabeculae are absent; (8) the cortical porosity (CP) of hyperelongated neural spines, a parameter used in previous publications for *D. grandis* and *D. giganhomogenes*^26^, as well as the unnamed *Dimetrodon* species of the Richards Spur locality^4^. CP is expressed in % as the ratio between the area occupied by vascular canals within the cortex and the area of mineralized cortical bone; (9) the outer circumference of the humeral and femoral cross-sections (Circ) in order to estimate the body mass of the diminutive *Dimetrodon* species based on the equations of^29^ and compare our results to the body mass estimates gathered by^2^ based on vertebral dimensions.

## Supplementary Information

Below is the link to the electronic supplementary material.

Supplementary Material 1

## Data Availability

The complete datasets generated and/or analyzed during the current study are available from the corresponding author on reasonable request. Full-resolution transverse section renders of all investigated *Dimetrodon* specimens are also available in the MorphoSource project ID 000850132 at: https://www.morphosource.org/projects/000850132?locale=en.
