# Supplementary material for: Contrasting life history in the diminutive Dimetrodon species from North America and Germany
Source: Sci Rep. 2026 Jun 16;16:16279. doi: 10.1038/s41598-026-52199-y (PMC13272913; doi:10.1038/s41598-026-52199-y)
Supplement: Supplementary file 1 — Supplementary Material 1 [file 41598_2026_52199_MOESM1_ESM.docx]

**Supplementary Information 1**

**Canoville et al.** - Contrasting life history in the diminutive *Dimetrodon* species from North America and Germany

This document contains:

1°) a figure comprising various trace fossils indicative of fossorial habits of Bromacker tetrapods and the unique depositional paleoenvironment at the Bromacker locality (Fig. S1).

2°) the bone microstructural descriptions and associated illustrations (Figs. S2 and S3) of all North American *Dimetrodon* specimens studied here. We reinvestigated previously made thin-sections (from the studies of Shelton et al., 2012 and Knaus, 2014), but also processed new slides of *Dimetrodon* material recovered from different North American localities, including marginal marine and more inland paleoenvironments (see Table 1).

**1°) Depositional paleoenvironment and fossil evidence of fossoriality at the Bromacker locality (Tambach Formation, lower Permian, Germany)**


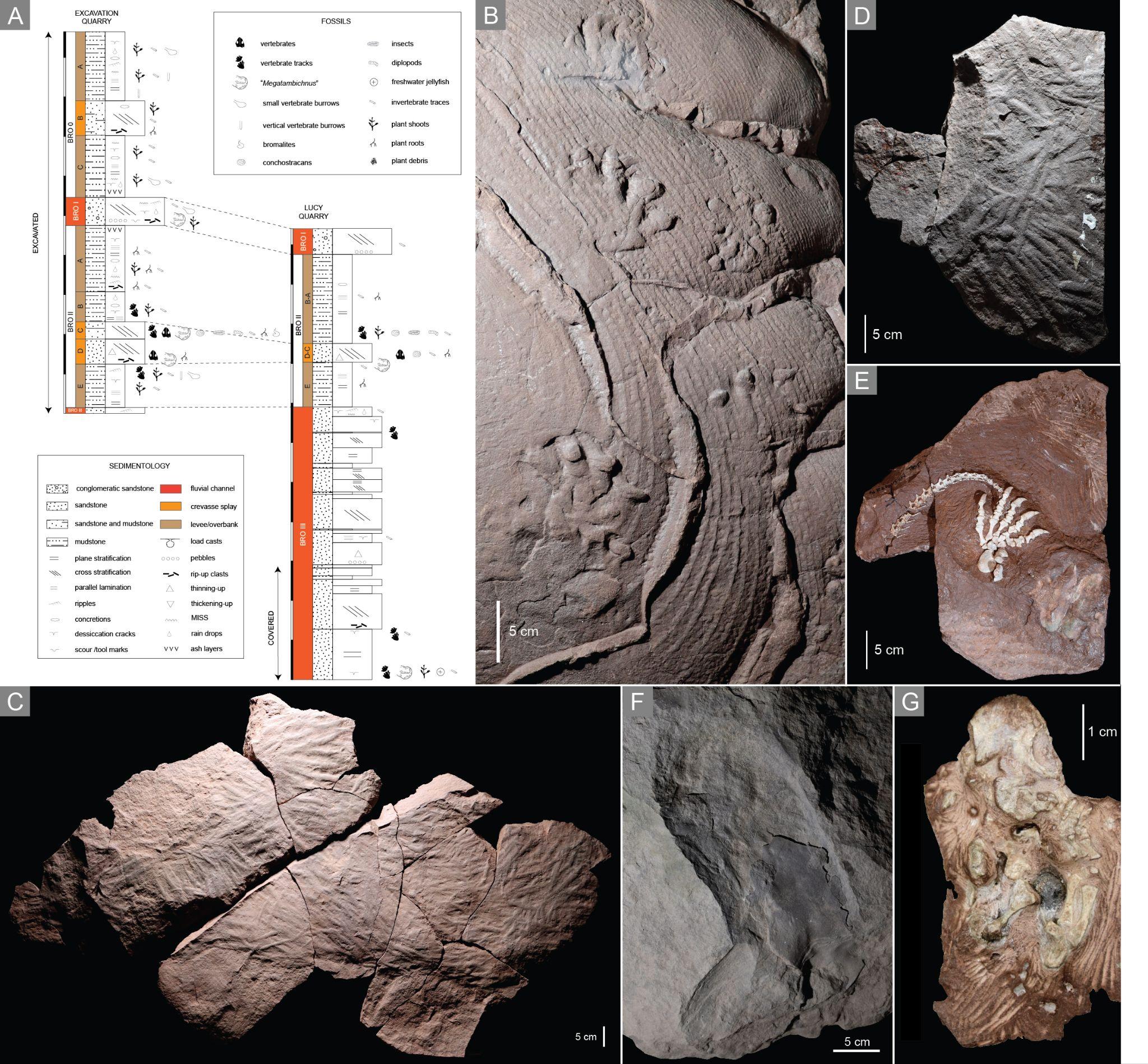


**Figure S1.** **Depositional paleoenvironment and fossil evidence of fossoriality at the Bromacker locality (Tambach Formation, lower Permian, Germany).** **A:** Stratigraphic log of the Bromacker locality, metric scale. **B:** Museum für Naturkunde Chemnitz MNC-F 1393. Contour marks, mud cracks and trackway of *Ichniotherium sphaerodactylum*, convex hyporelief. **C:** MNG 17118. Large sub-horizontal vertebrate burrow with scratch traces on the bottom, convex hyporelief, “*Megatambichnus*” isp. **D:** MNG 13814. Scratch traces on the bottom of the burrow infill, “*Megatambichnus*” isp., convex hyporelief. **E:** MNG 13814. Articulated tail and left pes of the *Martensius bromackerensis* holotype preserved in the burrow infill of D, dorsal view. **F:** MNG 16753. Additional vertebrate burrows. Horizontal burrow cut by a smaller oblique burrow on the side, concave epirelief. **G:** MNG 16545. The fossorial recumbirostran *Bromerpeton subcolossus*, holotype, dorsal view.

**2°) Bone microstructural descriptions and illustrations of North American *Dimetrodon* species**

**D. natalis *long bone and neural spine histology***

We reinvestigated the histology of *D. natalis* for comparison purposes with its diminutive relative *D. teutonis* (Fig. S2). The juvenile humerus IPBSH-13 (58 mm in length and only 48% of adult size according to Shelton et al. 2012) has a sub-circular cross-section at midshaft (Fig. S2A). The expansion of the medullary cavity was in progress, and the ontogenetically older (deeper) periosteal cortex was being resorbed at the time of death (Fig. S2A, C). Hence, the transition between the compact cortex and the medullary region is poorly defined (Fig. S2A, C), the overall cross-section is rather compact, and the bone wall is thick (RBT of 19.6%). As already described in Shelton et al. (2012), most of the cortex comprises a highly vascularized and uninterrupted woven-parallel complex (Fig. S2C). Primary osteons are either radial or longitudinal and organized in radial rows (Fig. S2C), attesting of a relatively high growth rate in this young animal. Besides, numerous vascular canals are still piercing the periosteal surface, testifying that diametrical growth was ongoing. Remodeling is very limited and secondary osteons are virtually absent in the compact cortex. Humerus IPBSH-4 has reached adult size (120 mm in length) and differs microstructurally from IPBSH-13. Its cross-section is sub-triangular (Fig. S2B) and relatively compact (Cg = 0.70). Although filled with a dense network of secondary trabeculae (S = 0.116), the medullary region is well-defined and its transition with the compact cortex conspicuous (Fig. S2B, D). The cortex remains relatively thick (RBT = 19.6%; CDI = 0.38) and is mostly formed of a highly vascularized woven to parallel-fibered tissue interrupted by several growth marks (Fig. S2D). Vascularization consists of long radial canals, but also longitudinal primary osteons organized in radial rows or interconnected by radial anastomoses (Fig. S2D). There is a marked decrease in vascularization and a more parallel-fibered structure of the tissue in the outermost cortex, indicating a clear slowdown in growth and the onset of an EFS (Fig. S2D). Remodeling is restricted to the deepest cortex and the trabecular network.

The smaller femur IPBSH-19 (98 mm in length) belonged to an individual 72% of adult body size. Its mid-diaphyseal cross section is oval (Fig. S2E). The compact cortex (RBT of 16.5%) is relatively thick and its transition with the open medullary cavity is well-defined. Again, the periosteal cortex consists in a well-vascularized woven to parallel-fibered tissue interrupted by poorly defined growth marks (Fig. S2G). The vascularization that reaches the periosteal surface combines radial canals and longitudinal primary osteons organized in radial rows, sometimes connected by radial anastomoses (Fig. S2G). Remodeling is restricted to large erosion bays in the perimedullary region. IPBSH-2 represents a fully grown femur (137 mm in length) that has been damaged by diagenesis, such as it is crushed dorso-ventrally, parts of the bone wall are missing, and the medullary region is obscured by sediments and recrystallizations (Fig. S2F). It is clear, however, that the cortex was relatively thick and the transition with the open medullary cavity well-defined. The preserved cortex shows a well-vascularized parallel-fibered tissue interrupted by several LAGs whose spacing decreases rapidly towards the periphery. A well-defined EFS is present in the outermost cortex (Fig. S2H).

Finally, the neural spine of MB.R.6827.1-3 cf. *D. natalis* exhibits the traditional dumbbell shape in cross section (Fig. S2I). The lateral cortices are thick. The deep cortex is composed of a relatively poorly-vascularized woven-parallel complex. Vascularization consists of longitudinal primary and secondary osteons (Fig. S2J, K). From about mid-cortex up to the periosteal surface, the tissue grades into less vascularized to avascular parallel-fibered tissue interrupted by regular, but faint, growth marks. Large resorption cavities lined with several layers of endosteal lamellar bone mark the transition between the compact cortex and the medullary cavity (Fig. S2K). These histological features indicate that this small individual was already a late juvenile or even an adult at the time of death.


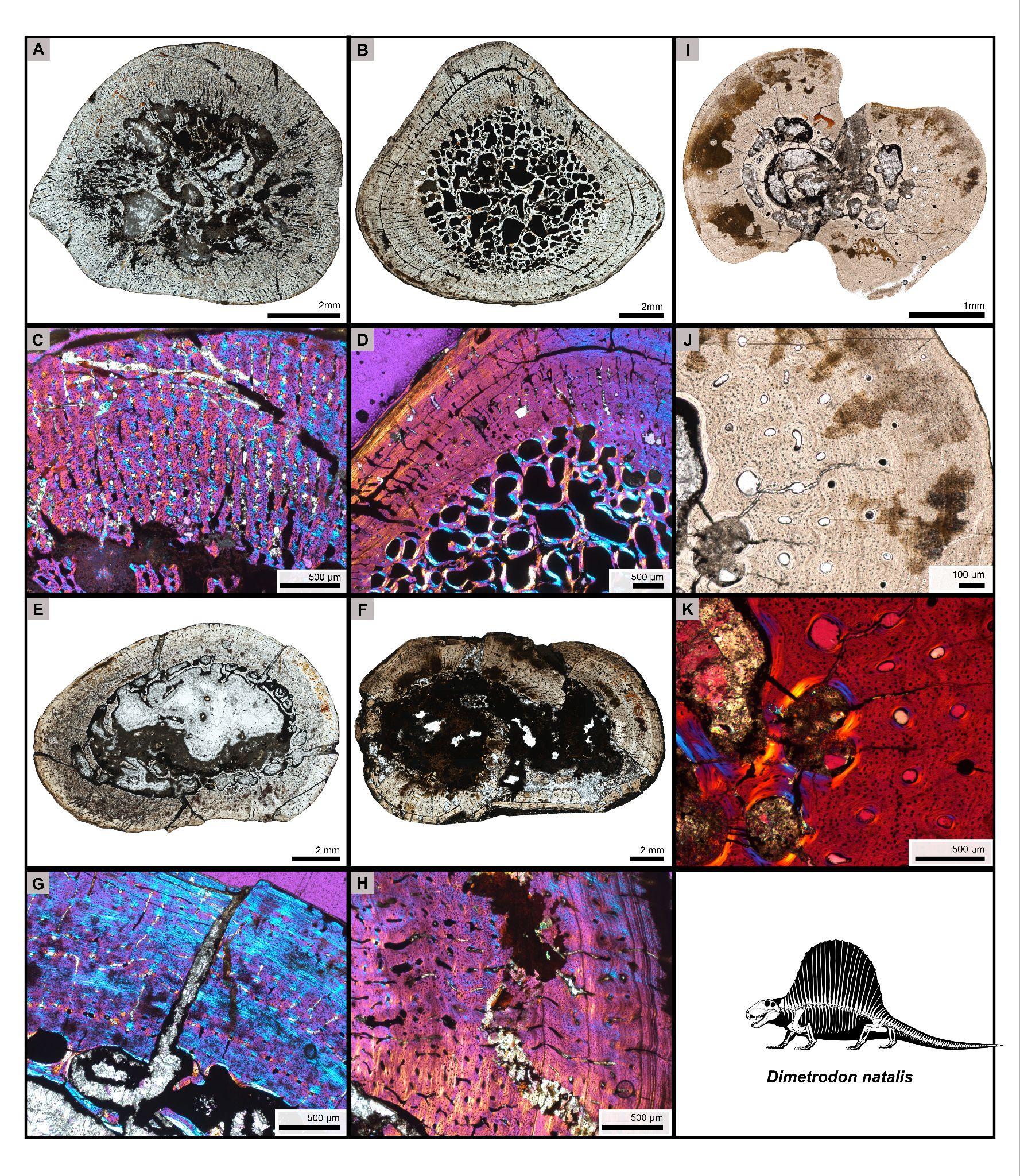


**Figure S2.** **Stylopod microstructure of selected *D. natalis* specimens.** **A:** mid-diaphyseal cross section of the humerus of juvenile specimen IPBSH-13; **B:** mid-diaphyseal cross section of the humerus of skeletally mature specimen IPBSH-4; **C:** close up of the cortex in (A). The periosteal cortex is formed of a highly vascularized and uninterrupted woven parallel-complex. Vascularization consists in longitudinal primary osteons arranged in radial rows and connected by radial anastomoses. The deep cortical bone is being resorbed; **D:** Close up of the compact cortex and medullary spongiosa in (B). The primary cortex is formed of a mostly well-vascularized parallel-fibered tissue interrupted by clear growth marks. Vascularization consists of longitudinal primary osteons, sometimes connected by radial anastomoses and arranged in radial rows, as well as simple radial canals. Vascularization markedly decreases in the outer cortex and an EFS is present. The medullary region is filled by a well-developed spongiosa made of secondary trabeculae; **E:** mid-diaphyseal cross section of the femur of juvenile specimen IPBSH-19; **F:** mid-diaphyseal cross section of the femur of skeletally mature specimen IPBSH-2; **G:** close up of the cortex and perimedullary region in (E). The periosteal cortex consists in a well-vascularized parallel-fibered tissue with longitudinal primary osteons organized in radial rows and connected by few radial anastomoses. Remodeling is restricted to the perimedullary region with large erosion bays bordered by a thin layer of endosteal lamellar bone and a few secondary trabeculae. Most of the medullary cavity is free of bone trabeculae. **H**: close up of the cortex in (F). The cortex shows a well-vascularized parallel-fibered tissue interrupted by several LAGs whose spacing decreases towards the periphery. An EFS is present close to the periosteal surface.

**D. giganhomogenes *long bone histology***

We also reinvestigated the femoral histology of three different-sized individuals (Fig. S3A-F) attributable to *D. giganhomogenes* (Knaus, 2014). The smallest femur OMNH 15044 (81 mm in length) has an oval cross-section at midshaft (Fig. S3A). The transition between its thick compact cortex (RBT of 16.3) and the medullary cavity comprising few trabeculae is abrupt. The cortex is composed of an uninterrupted and well-vascularized woven to parallel-fibered tissue (Fig. S3D). Vascularization is present up to the periosteal surface and mostly consists of longitudinal primary osteons organized in radial rows (Fig. S3D). Radial anastomoses are visible in localized areas. Remodeling is very limited and restricted to the endosteal margin and the few secondary trabeculae. The intermediate-sized femur TMM 30966-201 (142 mm in length) shows a thicker bone wall (RBT of 20.9%) that is very densely vascularized up to the periosteal surface and formed of an alternation of poorly defined zones (woven tissue) and annuli (parallel-fibered tissue) (Fig. S3E). Vascularization consists of longitudinal primary osteons arranged in radial rows without or with numerous radial anastomoses (Fig. S3E), depending on the area of the section. Remodeling is limited to the network of secondary trabeculae occupying the medullary region. The largest femur TMM 30966-49 (221 mm in length) shows a dense trabecular network of secondary trabeculae in the medullary region (Fig. S3C). The transition between the compact cortex (RBT of 18.2%) and the medullary territory is more gradual than in the two other femora (Fig. S3C). Its highly vascularized woven to parallel-fibered cortex comprises an alternation of zones and annuli of regular thickness (Fig. S3F). Again, depending on the area of the section, the numerous longitudinal primary osteons are just organized in radial rows or connected by radial anastomoses (Fig. S3F). Long radial canals are also present in parts of the section. Large erosion cavities are present in the deep cortex. Remodeling is restricted to the deep cortex and medullary region. Some vascular canals are still piercing the periosteal surface. However, three to four closely-spaced LAGs are present in the outermost surface and might represent the onset of an EFS (Fig. S3F).

**Dimetrodon *sp.* *long bone histology***

We also studied two *Dimetrodon* stylopods that could not be clearly assigned to a known species (Fig. 3G, H, J, K). Femur TMM 30966-291 (124 mm in length) has been hypothesized to belong to *D. natalis* or another diminutive species by Knaus (2014). Indeed, although slightly larger, this element shows similarities to femur IPBSH-19. Its cross section is oval with a RBT of 15.2% and a medullary cavity relatively open with only few trabeculae (Fig. S3G). The stratified cortex consists of well-vascularized zones made of woven to parallel-fibered tissue alternating with thinner and poorly vascularized parallel-fibered annuli, each associated with a LAG (Fig. S3J). Osteocyte lacunae are abundant throughout the section, but tend to be more organized and flattened within the annuli. Again, longitudinal primary osteons are connected by radial anastomoses (Fig. S3J). Simple radial canals are also numerous throughout the section.

Humerus OMNH 15060 has a sub-triangular cross-section along its shaft (Fig. S3H) and a relatively high global compactness (Cg = 0.80) and cortical thickness (RBT = 28.1%; CDI = 0.523), as seen in *D. natalis* IPBSH-4. The medullary region is filled by thick secondary trabeculae and remnants of incompletely resorbed older periosteal bone (Fig. S3H). The transition between the medullary region and the compact cortex is thus poorly defined and progressive (S = 0.122) . A large nutrient foramen penetrates the bone wall at this level of the shaft (Fig. S3H). The cortex consists of an alternation of highly to well-vascularized zones made of woven to a more parallel-fibered tissue towards the periphery, and thin parallel-fibered annuli sometimes associated with a LAG (Fig. S3K). The thickness of the zones and the density of vascularization markedly decrease towards the periphery (Fig. S3K), attesting of a decrease in depositional rate. Besides, longitudinal primary osteons arranged in radial rows are connected by long radial anastomoses in the deep cortex. The density of these anastomoses declines towards the bone surface (Fig. S3K). Finally, there is a clear EFS in the outermost cortex (Fig. S3K). This layer consists of a nearly avascular parallel-fibered tissue interrupted by several closely spaced LAGS. The histology and size of humerus OMNH 15060 thus confirms that this element belonged to a skeletally mature individual of a diminutive species.

**D. grandis *long bone histology***

We sampled one proximal (OMHN 15055a) and one distal (OMHN 15055b; Fig. S3I, L) humerus identified as juveniles of *Dimetrodon grandis*. The proximal section is oval, shows a thick compact cortex (RBT = 23.6%) and a transition with the medullary region relatively progressive due to the ongoing resorption of the deepest and oldest periosteal tissue. The distal section resembles a tear drop in shape (Fig. S3I), with a relatively thinner compact cortex (RBT = 17.7%) and a wider medullary region. As in the proximal section, the transition between the compact cortex and medullary region is progressive and poorly defined because of the resorption of the deepest cortex and the formation of secondary trabeculae (Fig. S3I). Despite some variations in overall microanatomy due to different section planes along the shaft, the histology of both sections is very similar, suggesting that they come from a young individual, and probably from the same skeletal element. In both sections, the periosteal tissue is made of a highly-vascularized woven to parallel-fibered tissue (Fig. S3L). Its deposition seems to have been uninterrupted up to the outermost cortex where a faint LAG marks the transition with a thin layer of less vascularized parallel-fibered tissue (Fig. S3L). Vascularization mostly consists of longitudinal primary osteons organized in radial rows and sometimes connected by radial or reticular anastomoses (Fig. S3L). Remodeling is high in the deep cortex and perimedullary region. The ontogenetically older cortical bone has been partly resorbed, as attested by the presence of large resorption cavities and erosion bays bordered by thin layers of endosteal lamellar bone. Remnants of primary periosteal tissue are progressively integrated into thick secondary trabeculae (Fig. S3L). Secondary osteons are also visible in the deep cortex.


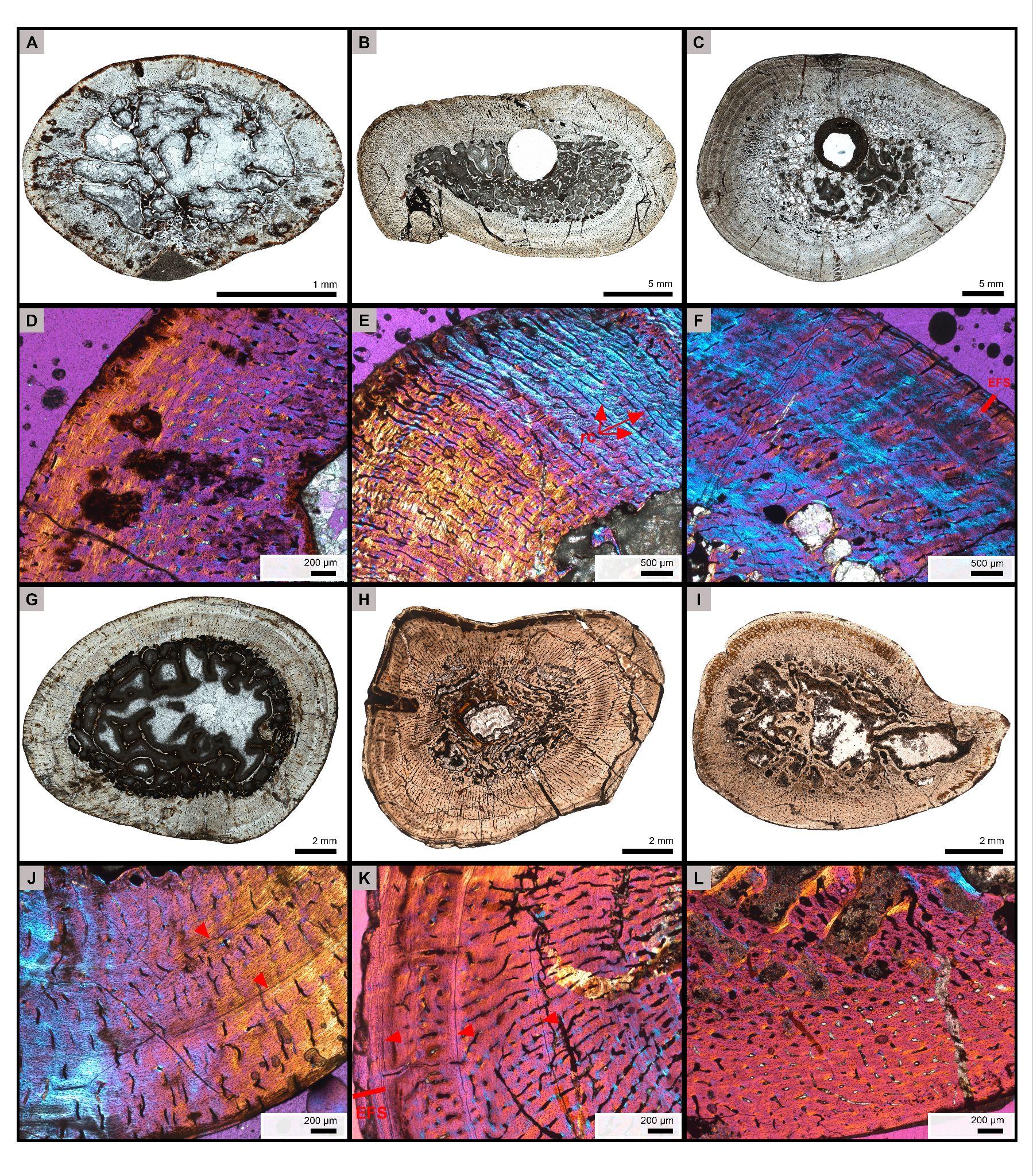


**Figure S3. Stylopod microstructure of selected NA *Dimetrodon* species. A:** mid-diaphyseal cross section of the small femur of *D. giganhomogenes* OMNH 15044; **B:** mid-diaphyseal cross section of the intermediate-sized femur of *D. giganhomogenes* TMM 30966-201; **C:** mid-diaphyseal cross section of the large femur of *D. giganhomogenes* TMM 30966-49; **D:** Close up of the periosteal cortex in (A). The cortex is formed of an uninterrupted and well-vascularized woven to parallel-fibered tissue. Vascularization consists of longitudinal primary osteons organized in radial rows, with few radial anastomoses; **E:** The cortex is very densely vascularized up to the periosteal surface. Vascularization consists of longitudinal primary osteons arranged in radial rows with numerous radial anastomoses and radial canals (rc). **F:** The cortex consists of a highly vascularized woven to parallel-fibered bone tissue with alternance of zones and annuli of regular thickness. The longitudinal primary osteons are organized in radial rows and sometimes connected by radial anastomoses. The onset of an EFS is visible in the outermost cortex; **G:** mid-diaphyseal cross section of the femur of *Dimetrodon* sp. TMM 30966-291; **H:** proximal shaft cross section of the small humerus of *Dimetrodon* sp. OMNH 15060; **I:** distal shaft cross section of the small humerus of *D. grandis* OMHN 15055; **J:** close up of cortex in (G). The stratified cortex is formed of well-vascularized zones made of woven to parallel-fibered tissue alternating with thinner and poorly vascularized parallel-fibered annuli, each associated to a LAG (red arrowheads). Longitudinal primary osteons are connected by radial anastomoses. Simple radial canals are also present; **K:** close up of the cortex in (H). The cortex consists of an alternance of highly to well-vascularized zones made of woven to a more parallel-fibered tissue towards the periphery, and thin parallel-fibered annuli sometimes associated with a LAG (red arrowheads). Longitudinal primary osteons arranged in radial rows are connected by long radial anastomoses in the deep cortex. The density of these anastomoses declines towards the periphery. An EFS is present in the outermost cortex; **L:** close up of the cortex in (I).
